# Successful Results of Intracytoplasmic Sperm Injection of a Chinese Patient With Multiple Morphological Abnormalities of Sperm Flagella Caused by a Novel Splicing Mutation in *CFAP251*

**DOI:** 10.3389/fgene.2021.783790

**Published:** 2022-01-11

**Authors:** Jiaxiong Wang, Ce Zhang, Hui Tang, Aiyan Zheng, Hong Li, Shenmin Yang, Jingjing Xiang

**Affiliations:** The Center of Reproduction and Genetics, Suzhou Municipal Hospital, The Affiliated Suzhou Hospital of Nanjing Medical University, Gusu School, Nanjing Medical University, Suzhou, China

**Keywords:** MMAF, ICSI (intracytoplasmic sperm injection), male infertility, CFAP251, uniparental disomy (UPD)

## Abstract

Asthenospermia is one of the most important causes of male infertility. Among asthenospermia, multiple morphological abnormalities of sperm flagella (MMAF) are relatively rare idiopathic conditions characterized by multiple defects in sperm flagella. Although many studies focusing on the genetic factors of MMAF have been conducted, its pathogenesis and treatment effect remain largely unknown. Here, we report a male patient from a nonconsanguineous Chinese family who exhibited a typical MMAF phenotype revealed by morphological analysis. We identified splicing mutations in *CFAP251* (c.1192-3C>G), and the mutation was proven to cause exon skipping. In addition, western blotting and immunofluorescence analysis of the spermatozoa from the proband and a control subject revealed a significantly lower expression of CFAP251 protein due to pathogenic mutation. Interestingly, the patient’s mother was a heterozygous carrier for the mutation, but his father was not, and finally, the inheritance pattern was proven to be maternal uniparental disomy. We applied an intracytoplasmic sperm injection and achieved a successful pregnancy. Above all, our findings expand the spectrum of *CFAP251* pathogenic mutations and provide more indications for clinical genetic counseling and assisted reproductive treatment for such patients.

## Introduction

Infertility affects approximately 15% of couples in the world, of which 20–30% are due to male factors, including asthenoteratozoospermia (Agarwal et al., 2015). Asthenoteratozoospermia can be caused by many factors, such as life habits and a history of chemical and radiation exposure. Interestingly, some asthenoteratozoospermia cases have shown uniform specific phenotypes in sperm flagella and are defined as a disorder of genetic origin, including primary ciliary dyskinesia (PCD) and multiple morphological abnormalities of sperm flagella (MMAF). Many genes have been identified to be associated with MMAF since the initial identification of *DNAH1* (Touré et al., 2021). However, the genetic causes and pathogenic mechanisms in many MMAF cases remain unresolved.

*CFAP251* (also termed *WDR66*, NM_001178003) is located on chromosome 12 and encodes cilia- and flagella-associated protein 251, which was first reported to be related to MMAF by Kherraf et al. (2018). The CFAP251 protein contains 14 WD repeat domains (WDRs) (Li et al., 2019) which also exist in the MMAF-related proteins CFAP43 and CFAP44 (Tang et al., 2017; Coutton et al., 2018). Among the 14 WDRs, nine are WD-40 repeat domains related to protein–protein interactions (Smith, 2008), and a calcium-binding EF hand domain is located at the carboxy-terminal extremity, which is important for flagellar structure and beating (Kherraf et al., 2018). Nine mutations of *CFAP251* in seven cases were reported to be relevant to MMAF (Auguste et al., 2018; Kherraf et al., 2018; Li et al., 2019). However, the reports of novel mutations are still valuable, and the assisted reproductive outcome of the patients harboring *CFAP251* variants remains unknown.

In our research, we identified a novel homozygous splicing variant c.1192-3C>G in a patient with MMAF by whole-exome sequencing (WES) from a large cohort of 32 asthenoteratozoospermia affected Chinese men. The subject harboring a homozygous splicing variant in *CFAP251* displayed a severe reduction in sperm motility and a typical MMAF phenotype. The mode of inheritance was proven to be maternal uniparental disomy (UPD). It was gratifying that the pregnancy outcome was good after the intracytoplasmic sperm injection (ICSI) treatment using the sperm from the patient with the *CFAP251* splicing variant. This finding expands the spectrum of pathogenic *CFAP251* variants that are associated with MMAF and male infertility.

## Materials and Methods

### Subjects and Clinical Investigation

The patient was one in a cohort of 32 subjects affected by MMAF enrolled at the Affiliated Suzhou Hospital of Nanjing Medical University. All recruited individuals displayed isolated infertility without other cilia pathological symptoms, such as bronchitis or sinusitis. The proband in this study displayed normal external genitalia and bilateral testicular development and hormone levels. Furthermore, all individuals had normal somatic karyotypes (46, XY) with no large-scale deletions found in the Y chromosome. Informed consent was obtained from all of the subjects and their family members participating in the study. This study was approved by the Ethics Committees of the Affiliated Suzhou Hospital of Nanjing Medical University.

### Semen Characteristics Analysis

After 2–7 days of sexual abstinence, semen samples were obtained by masturbating, and analyzed according to the 5th World Health Organization (WHO) guidelines, in our andrology laboratories. The analysis of semen volume, sperm concentration and motility was repeated twice. Hematoxylin and eosin (H&E) staining was used to evaluate sperm morphology, which divided into six categories: normal, absent, short, bent, coiled flagella and flagella of irregular caliber. Each subject must be examined at least 200 sperm to assess the percentage of abnormal sperm morphology.

### Electron Microscopy Evaluation

For transmission electron microscopy (TEM) observation, we obtained fresh sperm specimens by centrifuging at 400 g × 15 min, washing twice in PBS, and fixing in 2.5% phosphate-buffered glutaraldehyde. Then, use graded ethanol (50, 70, 90 and 100%) and 100% acetone for dehydration, and then infiltrate with 1:1 acetone. The specimens were immersed and embedded in Epon 812, sliced on an ultra-thin microtome, stained with uranyl acetate and lead citrate, and then observed and photographed with a TEM (TECNAI-10, Philips) 80 kV accelerating voltage.

### Whole-Exome Sequencing and Bioinformatic Analysis

We obtained Genomic DNA from extracting blood samples by using the DNeasy Blood & Tissue Kit (Qiagen,Germany). With IDT xGenExome Research Panel V1.0 (Integrated DNA Technologies) weprepared whole-exome sequencing of samples. The Qubit 2.0 Fluorometer (Thermo Fisher Scientific) evaluated the number of sequencing libraries. 2100 Bioanalyzer High Sensitivity DNA Assay (Agilent Technologies)was used to measure the quality and size of libraries. For next-generation sequencing, the qualified libraries were subjected to 2 × 150-bp paired-ends sequencing on the Illumina NovaSeq platform (Illumina, San Diego, United States). FASTQ files were aligned to the human reference genome (hg19/GRCh37) by BWA v0.7.13 (Li and Durbin, 2009). Variants (single nucleotide variants and indels) were genotyped from recalibrated BAM files by GATK 4.0 and annotated using ANNOVAR against multiple databases, including HGVS variant description, population frequency, disease or phenotype and variant functional prediction. Variants were classified as pathogenic, likely pathogenic, variant of unknown significance (VUS), likely benign, or benign following the American College of Medical Genetics (ACMG) guidelines (Richards et al., 2015). Copy number variations were detected by DNA copy R package 5, filtered and classified by ACMG guidelines and manually checked by using the Integrative Genomics Viewer. Confirmation of the mutation of the proband and familial cosegregation was used for Sanger sequencing.

### SNP Array

SNP-array analysis was performed on the AffymetrixCytoScan platform (Affymetrix, Santa Clara, CA, United States) as described previously (Xiang et al., 2020). UPDtool was used to classify isodisomy or heterodisomy from SNP-array data in parent-child trios (Schroeder et al., 2013).

### Reverse-Transcription PCR and TA Cloning Sequencing

To analyze the expression of the *CFAP251* gene, total RNA of human spermatozoa was extracted using the RNeasyPlus Micro Kit (QIAGEN), and approximately 300 ng of obtained RNA was converted into cDNA using the HiScript III first Strand cDNA Synthesis Kit (Vazyme, R312). RT-PCR was performed in a 25-μl mixture containing 10 μl of Rapid Taq Master Mix (Vazyme, p222), cDNA templates (17.5 ng), and oligonucleotide primers (10 pM each) in 35 cycles. After analysis by agarose-PAGE, the results were examined and recorded using a gel imager (Tanon 4600). 18S was analyzed in parallel as a reference. The primers for RT-PCR are listed in Supplementary Table S2. The RT-PCR products were directly cloned into the pTG19-T vector and sequenced.

### Western Blotting

For western blotting analysis, fresh semen was washed three times with PBS by centrifugation (3,000 g × 5 min). The supernatant was discarded, and the protein denaturation progress was conducted with 1 mM loading buffer at 100°C for 15 min. The proteins were separated using SDS-PAGE and electroblotted on PVDF membranes at 200 mA for 1 h at 4°C. The membrane was then blocked with 5% bovine serum albumin solution (A8020; Solarbio, China) for 1 h at room temperature before it was incubated overnight at 4°C with primary antibodies (anti-CFAP251, 1:1,000 dilution; Abnova, Taipei, China) (anti-GAPDH, 1:1,000 dilution; Sigma). Secondary antibodies (goat anti-mouse IgG, 1:3,000 dilutions; ProteinTech Group, China) were used to detect antigen content.

### Immunofluorescence

The immunofluorescence test was carried out according to a protocol described previously (Wang et al., 2021). The sperm were washed three times with PBS, smeared onto glass slides and air-dried. The sperm slides were next fixed with 4% formaldehyde in PBS at room temperature for 20 min, followed by washing in PBS three times. Slides were then blocked in 5% bovine serum albumin (A8020; Solarbio, China) and incubated overnight at 4°C with primary antibody anti-CFAP251 (H00144406-B01P, Abnova, China) and HRP-conjugated secondary antibody (GB23301, Servicebio, China). Furthermore, incubation was carried out at room temperature for 10 min with Cy3-tyramide (G1223, Servicebio, China), followed by washing with PBS three times. Then, the slides were incubated with anti-α-tubulin antibodies (GB12200, Servicebio, China) and HRP-conjugated secondary antibody followed by FITC-tyramide (G1222, Servicebio, China). The slides were counterstained with 5 mg/ml DAPI (G1012, Servicebio, China) and mounted with mounting media (G1401, Servicebio, China). Finally, fluorescence images were taken using a confocal microscope (Nikon Eclipse CI, Nikon, Japan).

### Intracytoplasmic Sperm Injection, Embryo Culture, Embryo Transfer and Time-Lapse Monitoring

The patient’s wife underwent a routine antagonist regimen to induce ovulation. Recombinant human follicle stimulating hormone (MerckSerono) was injected 2 days after menstruation. The hormone level test and ultrasound were carried out after 3–5 days. When the diameter of the follicle was 13–14 mm, 0.25 mg/d gonadotropin releasing hormone antagonist (GnRH-ant, Merck & Co., Ltd.) was injected. When the diameter of the dominant follicle reached 18 mm, 0.2 mg oftriptorelin acetate (Ferring Pharmaceuticals) was injected, and after 35 h, the oocytes were retrieved. The obtained cumulus oocyte complex was washed and placed in a protein-containing fertilization fluid (Vitrolife, Sweden), followed by ICSI after 5 h.

The fertilized 2PN embryos were placed in an Embryoslide petri dish (Vitrolife, Denmark) and then placed in an EmbryoScope time-lapse incubator (Vitrolife, Denmark) for culture. The shooting interval was set to 10 min, and continuous shooting was performed for 6 days.

## Results

### Multiple Morphological Abnormalities of Sperm Flagella Phenotypes in Patients

**FIGURE 1:**
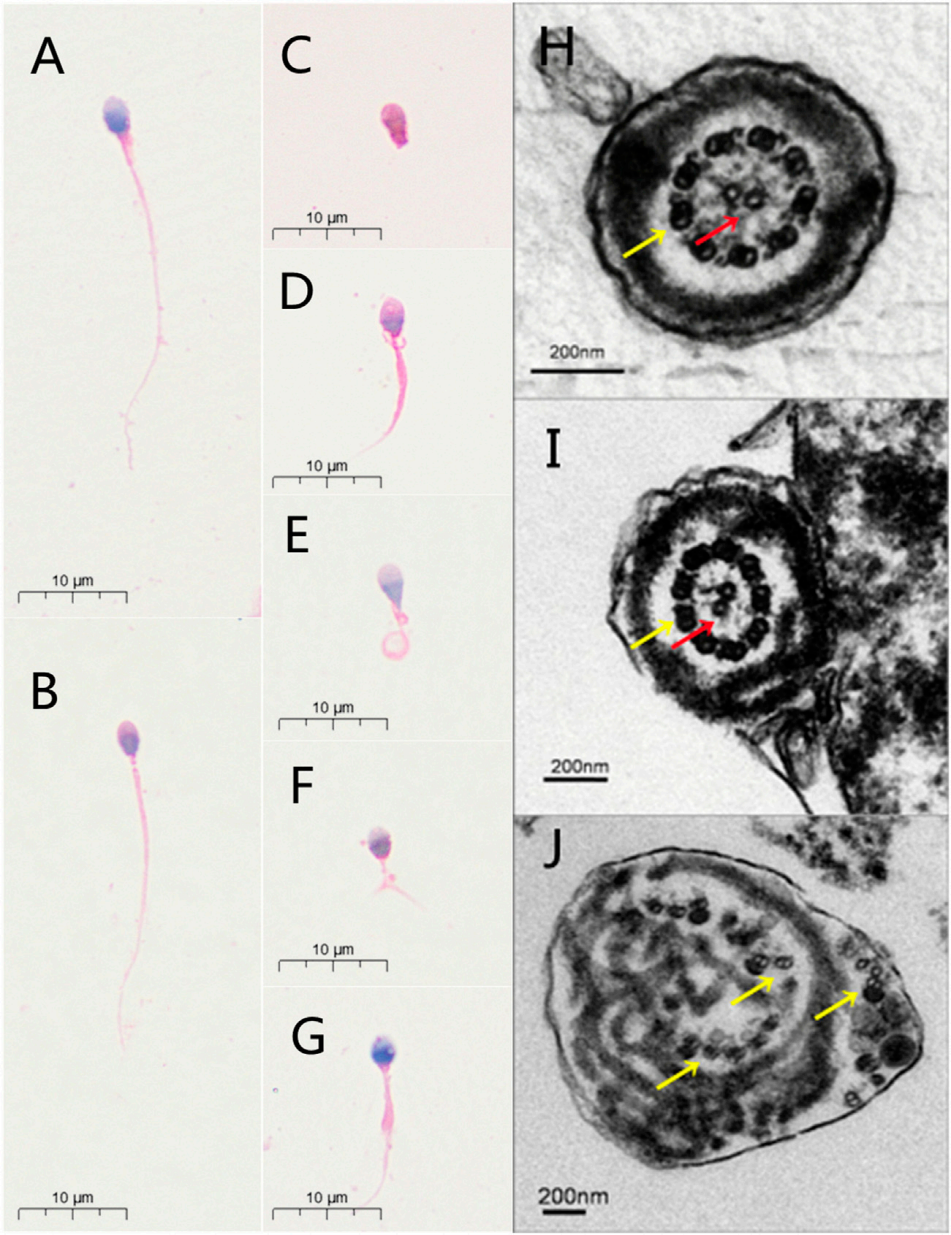
Sperm morphology analyses for men with *CFAP251* variant. Normal morphology of spermatozoa from a healthy control male as revealed by light microscopy **(A)**. The observed normal length of the sperm from a man harboring homozygous *CFAP251 sp*licing variant **(B)**. In addition, multiple malformations can be observed, including absent **(C)**, short **(D)**, coiled **(E)**, bent **(F)** and irregular caliber flagella **(G)**. Under TEM, the cross section of the normal sperm and very few sperm from men with *CFAP251* variant show a typical “9+2” structure of microtubules and the regularly arranged outer dense fibrous sheath **(H,I)**. Most of the sperm from the men with *CFAP251* variant show a severe disorder in the arrangement of the flagellar ultrastructure **(J)**. Central pair of microtubules (red arrows); peripheral microtubule doublet (yellow arrows).

Semen analysis indicated normal semen volume and sperm concentration, with severe reductions in sperm total and progressive motility in the patient (Table 1). Sperm morphological study under light microscopy showed the multiple malformations of the sperm flagella from the patient with the intronic *CFAP251* variant, including absent, short, coiled, bent and irregular flagella. However, the sperm head was normal. The rates of absent and coiled flagella were obviously higher in men with *CFAP 251* variant than in normal control (Table 1). Furthermore, under TEM, for sperm flagella, the cross sections of the patient with the *CFAP251* variant displayed typical severe disorganization in axonemal and other periaxonemal structures, in contrast to the “9+2” microtubule structure in normal males. It was worth noting that the patient with the *CFAP251* variant still had a few normal flagella, regardless of light microscope or electron microscope observation (Figure 1).

**TABLE 1:** Semen characteristics and sperm morphology in men harboring *CFAP251* variant.

Indicators	1	2	Reference limits
Semen parameter			
Semen volume (ml)	2.3	1.9	1.5^a^
Sperm concentration (10^6^/ml)	24.1	18.4	15.0^a^
Motility (%)	0.6	0.5	40.0^a^
Progressive motility (%)	0.3	0.5	32.0^a^
Sperm morphology			
Absent flagella (%)	29.5	37	14.0^b^
Short flagella (%)	36.0	36.5	5.0^b^
Coiled flagella (%)	25.5	15.5	1.0^b^
Angulation (%)	2.5	3.5	17.0^b^
Irregular caliber (%)	3.0	4.5	13.0^b^

a Reference limits according to the WHO standards.

b Reference limits according the article we reported before (Liu et al., 2021b).

### Identification of the Splicing *CFAP251* Variant in Men With Multiple Morphological Abnormalities of Sperm Flagella

**FIGURE 2:**
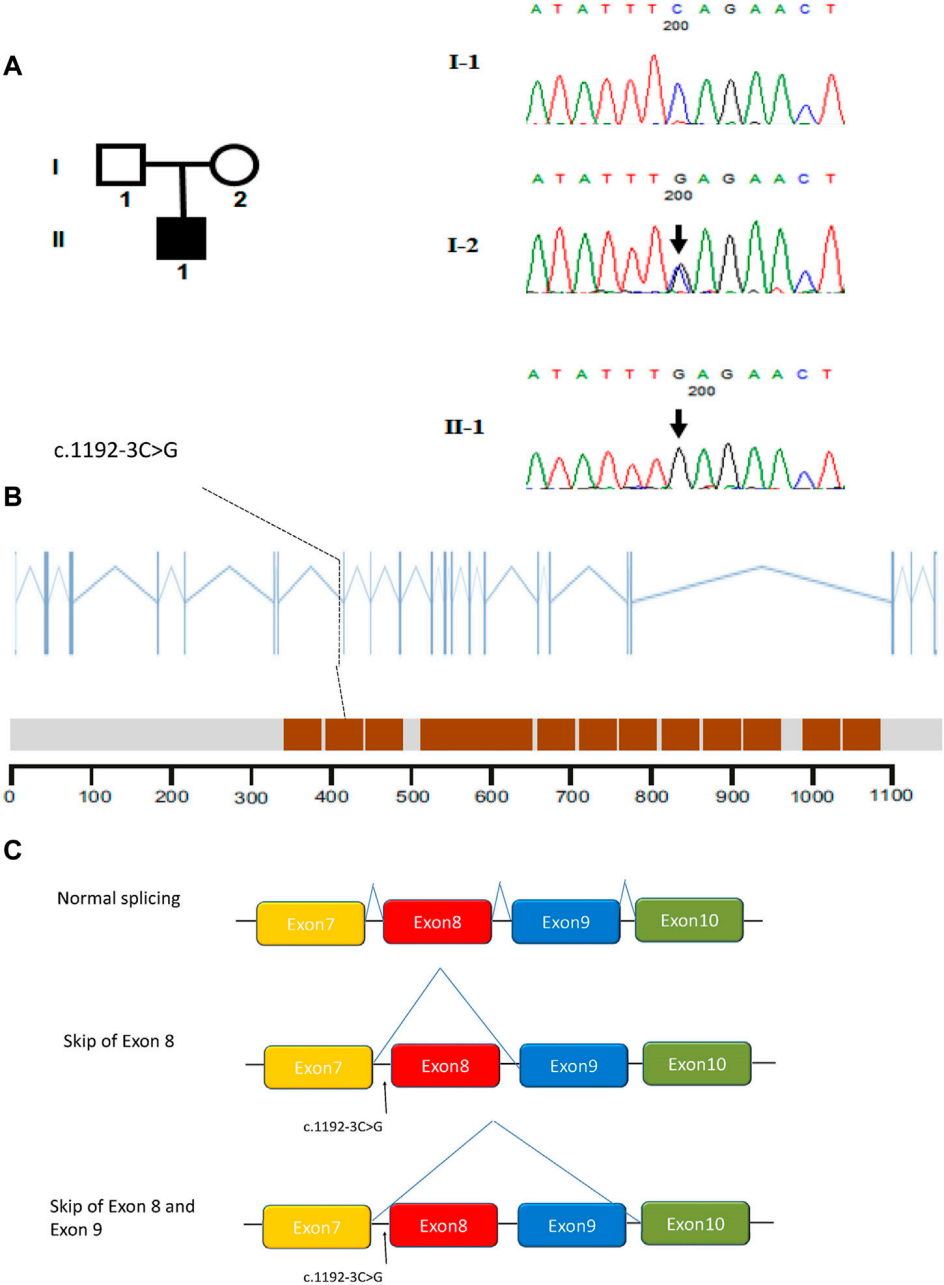
Identification of intronic variant of *CFAP251* in men with MMAF. **(A)** The pedigree of the family showing the father (I-1) and mother (I-2) with their offspring numbered II-1; black-filled squares indicate the male individuals with MMAF. Sanger sequencing results are shown below the pedigrees. The variant positions are indicated by black arrows. **(B)** The positions of the intronic *CFAP251* mutation are indicated in the *CFAP251* gene and the protein domains of *CFAP251*. The brown squares in the picture represent WD repeat domains. **(C)** Schematic representation of the impact of the splicing variant on mRNA splicing.

In this study, whole-exome sequencing analyses were performed on a cohort of 32 subjects affected by MMAF. Fortunately, we identified a splicing mutation in *CFAP251* (MIM: 614146): c.1192-3C>G. Interestingly, subsequent Sanger sequencing revealed that the patient’s mother carried the variant, but his father did not (Figure 2A). The location of the splicing mutation is shown in Figure 2B and was predicted to be related to the WD repeat domain.

**FIGURE 3:**
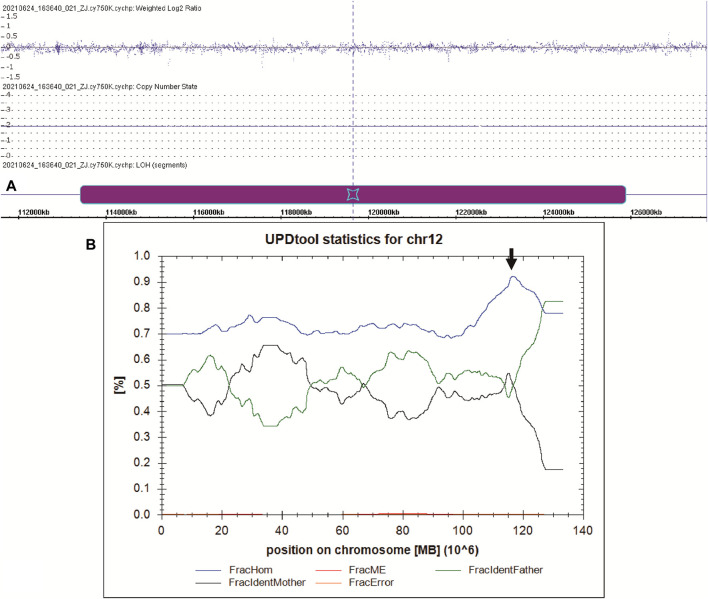
UPD analysis of the manharboring a *CFAP251* variant. **(A)** The SNP-array result of the patient. The purple box indicates the region of ROH on chromosome 12. **(B)** The results of UPD tool for the classification of uniparental disomy (UPD). Blue line indicates fraction of homozygous SNPs; red line indicates fraction of mendelian error SNPs; green line indicates fraction of SNPs where the genotype is identical to the father; black line indicates fraction of SNPs where the genotype is identical to the mother; yellow line indicates fraction of errors (=ME that cannot be explained by UPD). The black arrow indicates the region of isodisomy from 115 to 125 MB of chromosome 12.

To better understand the patient’s puzzling family inheritance, the WES data were reinvestigated, and we found that the 11 mutations in *CFAP 251* were all homozygous. This phenomenon indicated that the mode of inheritance was probably maternal uniparental disomy. A SNP array was carried out to verify this hypothesis, and a final12.461 Mb region of homozygosity (ROH) (arr [GRCh37] 12q24.13q24.31 (113436822_125897463)hmz), including *CFAP251*, was identified in the patient. UPDtool analysis for chromosome 12 of the patient and his parents revealed a high fraction of homozygous genotypes (>85%) identical to the mother, indicating an isodisomy from 115 to 125 MB of chromosome 12 (Figure 3).

### Novel Splicing Mutation *CFAP251* Leads to Exon Skipping in *CFAP251*

**FIGURE 4:**
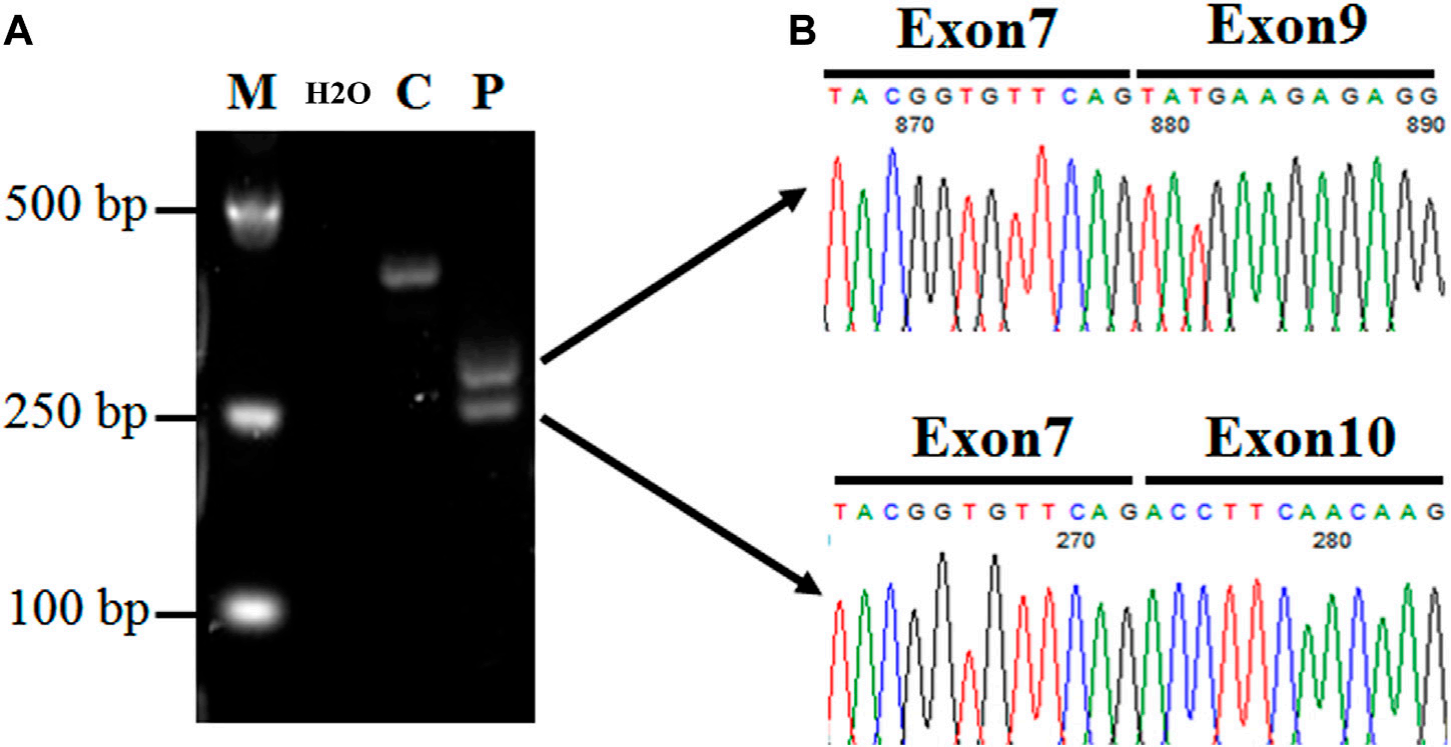
Expression analysis of *CFAP251* mRNA in spermatozoa from a normal male control and a man harboring the *CFAP251* variant. **(A)** The RT-PCR product obtained from the proband displayed two bands obviously shorter than the normal control, suggesting two splicing alterations. **(B)** The TA cloning sequencing results of the two electrophoresis bands show the skipping of exon 8 and both exons 8 and 9.

**FIGURE 5:**
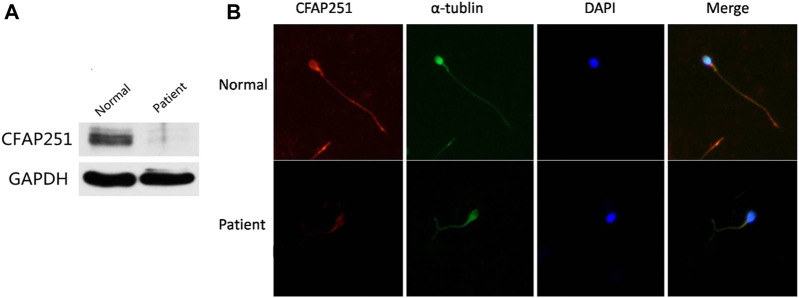
Expression and Location Analysis of CFAP251 Protein in Sperm. **(A)** Immunoblotting analysis of protein in sperm lysates from a normal control and from men harboring the CFAP251 mutation. GAPDH was used as a loading control. **(B)** The spermatozoa were stained with anti-CFAP251 (red) and anti-α tubulin (green) antibodies. Tubulin antibody staining indicates flagella, and DAPI staining indicates the nucleus of spermatozoa. Compared with the normal control, CFAP251 protein is absent from the sperm flagella of patients.

Since the mutation was located in the intronic area, further investigation of the pathogenicity of *CFAP251* variant was necessary. We obtained sperm RNA from a fertile control subject and from a patient harboring the *CFAP251* mutation and analyzed the expression of *CFAP251* mRNA using RT-PCR. The amplified reverse transcription product of sperm *CFAP251* RNA in the patient with the*CFAP251* variant showed two bands (308 and 257 bp) that were significantly shorter than the normal band (386 bp) in the electropherogram (Figure 4A). TA cloning sequencing showed that the novel splicing mutation in *CFAP251* led to two abnormal splicing events, which caused the skipping of exon 8 and exons 8 and 9, respectively (Figure 4B). Simultaneously, western blotting showed the absence of CFAP251 protein translation due to the splicing mutation, and CFAP251 immunostaining was mainly localized at the sperm flagella in normal spermatozoa but was almost absent in the sperm from patients with the *CFAP251* variant (Figure 5). These experimental results suggested the important contribution of the novel splicing *CFAP251* variant to MMAF.

### Good Outcome of Intracytoplasmic Sperm Injection in Patients With the Novel Splicing *CFAP251* Mutation

**FIGURE 6:**
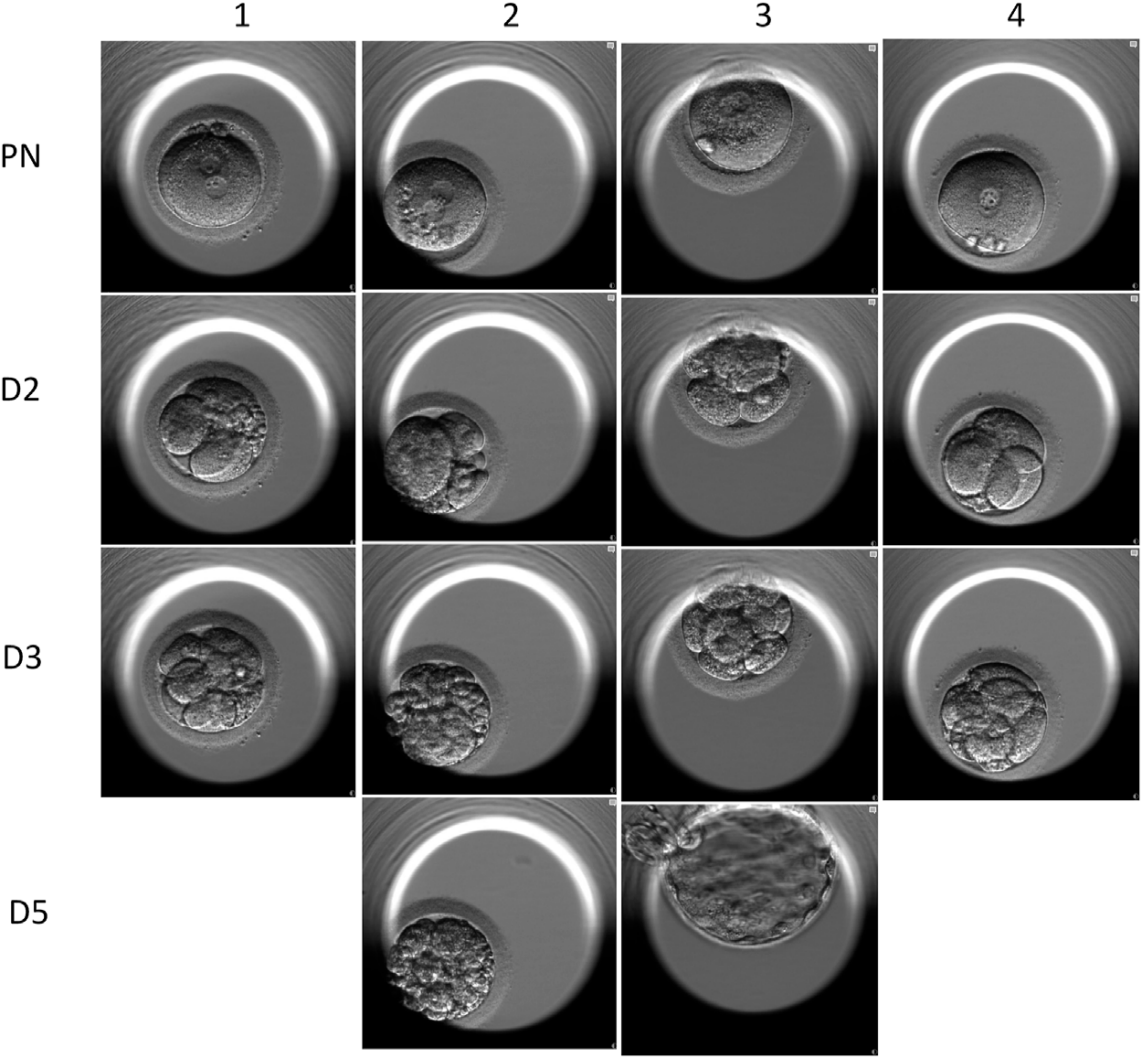
Time-lapse images of the embryos of the ICSI cycles. Line 1 shows that the four embryos all carried 2 PN. Line 2 shows the 2-day embryos, and the embryo scores were 421, 243, 512, and 411. Line 3 shows the 3-day embryos, and three of the four embryos were qualified as good quality. Line 4 shows the two transferred blasts.

On the oocyte retrieval day, only 3.2% motile sperm were found in the ejaculate. After the optimization program, the motility rate was raised to 4.8%. Sperm with comparatively normal morphology were injected into six oocytes. Four oocytes were fertilized, and finally, two blastocysts were transferred. The images of the embryos shot by the Time-Lapse incubator are shown in Figure 6. Successful pregnancy was confirmed by a positive result of the serum β-hCG test 14 days after transplantation, and a visible gestational sac was observed by ultrasound 4–6 weeks later. The ICSI results are summarized in Table 2.

**TABLE 2:** Clinical outcomes of ICSI cycles using spermatozoa from men with homozygous *CFAP251* splicing variant.

Subject	1
Male age (year)	30
Female age (year)	29
Number of ICSI cycles	1
Number of oocytes injected	6
Number (and rate) of fertilized oocytes	4 (66.67%)
Number (and rate) of cleavage embryos	4 (100%)
Number (and rate) of high-quality embryos	3 (75%)
Number of transfer cycles	1
Number of embryos transferred per cycle	2
Implantation rate	50%
Clinical pregnancy rate	50%
Miscarriage rate	50%

## Discussion

Flagellogenesis is an important biological process involving multiple organs, such as sperm, respiratory tract, oviduct, and myelocoele, and is regulated by complex mechanisms (Diniz et al., 2012). During this process, many proteins play important roles. MMAF, also termed dysplasia of fibrous sheath (DFS) (Chemes et al., 1987), is a type of dysfunction in flagellogenesis that has been reported to be related to several genes, such as *DNAH1* (Ben Khelifa et al., 2014), *CFAP43, CFAP44* (Tang et al., 2017), *TTC21A* (Liu W. et al., 2019), *TTC29* (Liu C. et al., 2019), etc. Here, using WES on a cohort of 32patientswith MMAF, we identified a novel splicing mutation in *CFAP251*. Prior to our study, two splicing mutations in *CFAP251* (c.2862+1G>A;c.1286 + 2 T > C) were reported (Kherraf et al., 2018; Li et al., 2019). Our results showed that the novel splicing mutation c.1192-3C>G in *CFAP251* resulted in exon skipping, and the c.1192-3C>G variant was not recorded in the dbSNP (http://www.ncbi.nlm.nih.gov/SNP/), Exome Aggregation Consortium (ExAC) (http://exac.broadinstitute.org/), Genome Aggregation Database (gnomAD) (http://gnomad.broadinstitute.org/) or 1000 Genomes Project database (http://www.1000genomes.org/).According to the ACMG variant classification guideline (Richards et al., 2015), the c.1192-3C>G variant could be classified as likely pathogenic with 1 strong (PS3) and 2 supporting (PVS1_Moderate, PM2_Supporting) evidences. Therefore, the MMAF phenotypes in this case are likely to be caused by splicing variant in *CFAP251*.

*CFAP251* is a gene containing 23 exons located on chromosome 12q24.31 and was first identified by Kherrafet al. (2018) and it is a member of the WD repeat-containing family that is highly expressed in the testis and, at lower levels in the lung, and very low levels have been detected in the kidney and brain. Previous studies indicated that CFAP251 was mainly located at sperm flagella, which was proven by our fluorescence test. As we know, the intronic mutations sometimes caused the disorder of mRNA splicing, and further affected protein translation (Kole et al., 2012). Our results showed that the novel splicing mutation in *CFAP251* resulted in exon 8 or exon 8, 9 skipping, causing errors in protein translation. According to the location of the variant, specific changes in the protein may affect the function of the WD repeat domain and cause flagellar assembly abnormalities.

Several studies have reported the association of *CFAP251* with MMAF and male infertility. Consistently, in our study, spermatozoa from men harboring *CFAP251* mutation also displayed multiple malformations in flagella, including higher rates of absent and short flagella. Ultrastructure analysis further revealed partial defects or loss of dramatic disorganization in axonemal or other periaxoneal structures in spermatozoa from men harboring the *CFAP251* splicing variant. It is worth noting that not all the flagella of the patient were abnormal, which made the patient’s sperm retain a certain amount of motility. In the study of Auguste et al. (2018) the sperm motility of the patients harboring *CFAP251* variant showed a total absence, while the other two reports were similar to our study: several patients’ sperm lacked total motility, and some retained partial motility (Kherraf et al., 2018; Li et al., 2019). Thus, our study combined with previous findings fully confirms that *CFAP251*is an important candidate gene for MMAF, but the correlation between specific phenotypes and different variants still needs more cases and statistical analysis.

Uniparental disomy is defined as two copies of a whole chromosome or homologous chromosomal segment derived from the same parent and is rare in an apparently healthy population and in spontaneous abortion tissues (Benn, 2021). The most common type of UPD is a maternal heterodisomy. Thus far, it has been reported that the inheritance mode of the pathogenic genes in MMAF is recessive, the mutations are homozygous or compound heterozygous (Touré et al., 2021), and no *CFAP251* UPD has been reported in MMAF, which might lead us to neglect genetic counseling; that is, as long as the spouse of the patient was not the carrier, the offspring would not be affected. In our study, the mother of the probands carried the heterozygous variant, while the father was normal, which confused us in terms of understanding the underlying mechanism of this situation. We investigated the CNV and microdeletion in *CFAP251*of the patient’s father by quantitative PCR and found nothing of note. Finally, the findings of the mutations in *CFAP251* of the proband were homozygous, indicating the possibility of UPD, which was proven by SNP array which is an effective approach to verify UPD. When UPD occurred, the most appropriate follow-up testing should consider the possibility of consanguinity which will generally involve multiple regions, an imprinted gene disorder (chromosomes 6, 7, 11, 14, 15, 20), expression of an autosomal recessive disorder (Benn, 2021). In our case, the parents of the proband were not consanguinity, the *CFAP251* located on chromosome 12, and the variant in the proband’s mother was heterozygous, which led our judgment more inclined to the third situation. The mechanism of UPD involves several processes, including meiotic non-disjunction with mitotic correction, gametic complementation, mitotic segregation error, or micronucle (Conlin et al., 2010). Here, we propose that genetic analysis of related genes should be carried out in the offspring of MMAF patients obtained by assisted reproduction to prevent the occurrence of UPD and the impact on fertility of the offspring.

For MMAF patients, assisted reproductive technology, ICSI, has becomes an important, even only way to achieve a successful pregnancy. Several studies and our previous works have revealed the outcomes of ICSI for a series of MMAF-related genes. In these studies, most of the patients with defects in different genes acquired good clinical outcomes, including, for example those with*DNAH1* (Sha Y. et al., 2017), *CFAP43, CFAP44* (Sha et al., 2019), *TTC29* (Liu C. et al., 2019) and *CFAP47* (Liu et al., 2021a) variants. Nevertheless, pregnancy failures have been reported, such as with *CEP135*, which could be due to the essential role of the sperm centrosome in the control of the first division after egg fertilization (Sha Y.-W. et al., 2017). Our study was the first ICSI outcome report of a patient with the *CFAP251* variant, and the results showed that successful clinical pregnancy could be achieved when the patient harboring the *CFAP251* mutation was treated with ICSI. This result was consistent with the pregnancy outcomes of most MMAF patients and indicated that ICSI was still the most suitable therapy for *CFAP251*-associated MMAF.

## Conclusion

In conclusion, we revealed a novel splicing mutation in *CFAP251*. Our experimental observations support the importance of *CFAP251* in the genesis of sperm flagella. The rare inheritance mode, maternal uniparental disomy, was first reported in MMAF patients, which indicated to us that even if only one parent was a heterozygous variant carrier, the offspring would still suffer a probable risk of MMAF. A good pregnancy outcome was achieved by ICSI treatment using spermatozoa from the patient with a *CFAP251* variant. Our findings provide new insight and further understanding of the genetic etiology and therapy of MMAF.

## Data Availability

The authors acknowledge that the data presented in this study must be deposited and made publicly available in an acceptable repository, prior to publication. Frontiers cannot accept an article that does not adhere to our open data policies.
