# Supplementary material for: Successful Results of Intracytoplasmic Sperm Injection of a Chinese Patient With Multiple Morphological Abnormalities of Sperm Flagella Caused by a Novel Splicing Mutation in CFAP251
Source: Front Genet. 2022 Jan 11;12:783790. doi: 10.3389/fgene.2021.783790 (PMC8787216; doi:10.3389/fgene.2021.783790)
Supplement: Supplementary file 1 [file Table1.DOCX]

Supplementary Material

**SupplementaryTable1Primers and Cycling conditionsUsed for Amplification and Verification of*CFAP251* Mutations.**

| **Primer** | | **Sequence** | | **Tm** | |
| --- | --- | --- | --- | --- | --- |
| *CFAP251-*F | | 5’-AGGGAACACATTTGACACTGT -3’ | | 58°C | |
| *CFAP251-*R | | 5’- CTCACCCCTCTAATCCCAGC-3’ | |  |  |
| **Program** | **Cycles** | | **Target(°C)** | | **Hold(hh:mm:ss)** |
| Initial-denature | 1 | | 95 | | 3 mm |
| Denature | 35 | | 95 | | 15 ss |
| Annealling |  |  | 55 | | 15 ss |
| Extension |  |  | 72 | | 30 ss |
| Final extension | 1 | | 72 | | 5 mm |

**SupplementaryTable2 Primers of the RT- PCR assay for*CFAP251* expression.**

| **Primer** | **Sequence** | **Tm** |
| --- | --- | --- |
| *CFAP251-*F | 5’-TGCCTCCTGATTATATGGGACT-3’ | 58.1°C |
| *CFAP251-*R | 5’-TCGCTAAACTTTCCCACAAGC-3’ | 59.9 |
| *18S-*F | 5’-GGAGTATGGTTGCAAAGCTG-3’ | 55.8°C |
| *18S-*R | 5’-CGCTCCACCAACTAAGAACG-3’ | 58.4 |
